# The Interaction between a Sexually Transferred Steroid Hormone and a Female Protein Regulates Oogenesis in the Malaria Mosquito *Anopheles gambiae*

**DOI:** 10.1371/journal.pbio.1001695

**Published:** 2013-10-29

**Authors:** Francesco Baldini, Paolo Gabrieli, Adam South, Clarissa Valim, Francesca Mancini, Flaminia Catteruccia

**Affiliations:** 1 Department of Immunology and Infectious Diseases, Harvard School of Public Health, Boston, Massachusetts, United States of America; 2 Dipartimento di Medicina Sperimentale e Scienze Biochimiche, Università degli Studi di Perugia, Terni, Italy; Stanford University, United States of America

## Abstract

Molecular interactions between male and female factors during mating profoundly affect the reproductive behavior and physiology of female insects. In natural populations of the malaria mosquito *Anopheles gambiae*, blood-fed females direct nutritional resources towards oogenesis only when inseminated. Here we show that the mating-dependent pathway of egg development in these mosquitoes is regulated by the interaction between the steroid hormone 20-hydroxy-ecdysone (20E) transferred by males during copulation and a female *Mating-Induced Stimulator of Oogenesis* (*MISO*) protein. RNAi silencing of *MISO* abolishes the increase in oogenesis caused by mating in blood-fed females, causes a delay in oocyte development, and impairs the function of male-transferred 20E. Co-immunoprecipitation experiments show that MISO and 20E interact in the female reproductive tract. Moreover *MISO* expression after mating is induced by 20E via the *Ecdysone Receptor*, demonstrating a close cooperation between the two factors. Male-transferred 20E therefore acts as a mating signal that females translate into an increased investment in egg development via a MISO-dependent pathway. The identification of this male–female reproductive interaction offers novel opportunities for the control of mosquito populations that transmit malaria.

## Abstract 2

Steroid hormones transferred by the male during sex trigger a molecular cascade of events that increases the reproductive success of females in *Anopheles gambiae* mosquitoes.

## Author Summary

*Anopheles gambiae* mosquitoes are the most deadly vectors of human malaria. The reproductive ability of these mosquitoes contributes to their role as disease vectors as it ensures high population densities for malaria transmission. The number of eggs developed by females after blood feeding depends on whether they have previously mated. Indeed in natural mosquito populations, virgin females rarely develop eggs when blood fed. Here we report on the identification of a molecular interaction between 20-hydroxy-ecdysone (20E), a steroid hormone transferred by the male during sex, and the Mating-Induced Stimulator of Oogenesis (MISO), a female reproductive protein, expression of which is triggered by mating and leads to increased egg production. We show that the expression of *MISO* after mating is regulated by 20E via the Ecdysone receptor (EcR). Experimental silencing of *MISO* reduces the ability of mated females to develop eggs after blood feeding, by reducing expression of a vitellogenic lipid transporter. By showing how male mosquitoes contribute to oogenesis in females, we identify a molecular pathway that can be targeted to reduce the reproductive success of natural mosquito populations to aid malaria control.

## Introduction

In many organisms, male–female molecular interactions occurring during sex shape reproductive success and may drive the rapid evolution of reproductive phenotypes [1]. While in species where females mate multiple times these reproductive interactions are often antagonistic due to the different reproductive strategies utilized by males and females 2–5, in monandrous species—that is, species where females mate a single time—they are believed to benefit both sexes [6]. Indeed this hypothesis has been proven experimentally in *Drosophila melanogaster*: removing sexual selection in this naturally promiscuous species through “imposed” monogamy induced the evolution of less antagonistic traits, where males became less harmful and females less resistant to induced harm [7].

In the malaria mosquito *Anopheles gambiae*, females rarely mate more than once during their lifetime [8]. As yet unknown male–female molecular interactions occurring during this single copulation regulate a series of postmating events that profoundly change the physiology and behavior of females. While *in copula*, females receive sperm, which are stored in a dedicated store organ named the spermatheca, and seminal secretions produced by the male accessory glands (MAGs). MAG secretions coagulate during mating to form a gelatinous mating plug that is transferred to the uterus (atrium), where it is digested in 1–2 d [9],[10]. Following this copulation event, blood-fed females increase their egg production [11] and start laying eggs [12],[13]. The regulation of egg production in *A. gambiae* is a particularly intricate process that depends on two main signals: one derived from blood feeding and one triggered by mating. While all females need to feed on blood to develop eggs, virgins in general have a pregravid state where they require two or more consecutive feedings to complete the first gonotrophic cycle 14–16. This has profound implications for malaria transmission, as it increases the likelihood of contact with the human host. Pregravid behavior may be caused by insufficient metabolic reserves at emergence due to nutritional deprivation during larval stages [14],[17]. This, in turn, may drive the need to optimize resource allocation between highly energy-demanding processes like flight and reproduction [18]. Indeed smaller *A. gambiae* mosquitoes tend to produce fewer eggs [19],[20] and appear to feed as virgins [21], perhaps to build up energy reserves for mating.

The cascade of events triggered by blood feeding and leading to egg development, partially described in *A. gambiae* [22], has been well characterized in another mosquito species, the yellow fever and dengue vector *Aedes aegypti*. In these mosquitoes, after a blood meal the ovaries are released from their previtellogenic arrest and start vitellogenesis, the process of synthesis and secretion of yolk protein precursors (YPPs) by fat body cells. Upon secretion into the hemolymph, the YPP Vitellogenin (Vg) and the lipid transporter Lipophorin (Lp) become internalized into the ovaries via receptor-mediated endocytosis [23],[24], leading to the maturation of 50–150 oocytes in approximately 2–3 d (reviewed in [25]). The transcription of YPPs is under endocrine regulation. After blood feeding the brain-secreted ovarian ecdysteroidogenic hormone (OEH) stimulates the ovaries to produce the steroid hormone ecdysone (E) [26],[27], which in turn is hydroxylated into 20-hydroxy-ecdysone (20E) by the fat body cells. 20E synthesis releases the state of arrest of the fat body, activating the transcription of YPPs [25],[28]–[30] by binding to the nuclear hormone receptor heterodimer Ecdysone Receptor (EcR)/Ultraspiracle (USP), prompting it to function as a transcriptional activator [31]. A similar role of 20E in vitellogenesis after blood feeding has been demonstrated also in *A. gambiae* [22], where titers of 20E in blood-fed females correlated to *Vg* expression, suggesting a conservation of this pathway between *Anopheles* and *Aedes* mosquitoes.

No information is instead available on the factors regulating the mating-induced stimulation of oogenesis observed in *A. gambiae*. Mating increases the rate of egg production in a number of insects, and in some cases this effect has been attributed to the transfer of MAG secretions (reviewed in [32]). The *D. melanogaster* Sex Peptide increases production of YPPs and oocyte maturation by inducing the female corpora allata to synthetize the sesquiterpenoid Juvenile Hormone III-bisepoxide (JHB3) [33]–[35]. In *Photinus* fireflies, seminal secretions translocated to ovaries positively influence female fecundity [36]. In mosquitoes, a role of MAG products in egg development has been suggested by a number of studies where injections of MAG extracts into the hemolymph of *Aedes* females stimulated Vg synthesis and/or oogenesis [37]–[40]. In *A. gambiae*, indirect evidence suggests that MAG secretions act as master regulators of female postcopulatory behavior and physiology [41]–[45]. Thus far more than 100 *A. gambiae* MAG genes have been identified [46],[47], and a number of them encode proteins that are packaged in the mating plug and transferred to females [10]. The *A. gambiae* MAGs, so far uniquely among mosquitoes, also produce large amounts of 20E [48], and delivery of this potent regulator of gene expression during sex may at least partly explain the vast transcriptional response that females undergo after mating [49]. This hypothesis is strengthened by the finding that among the genes regulated by mating is the 20E-responsive gene *Vg*, which is strongly induced in the female reproductive tract at 6 h after copulation [49].

Here we show that the 20E steroid hormone produced by the male and transferred to the female reproductive tract during copulation triggers a series of molecular events leading to the increased egg production observed in blood-fed *A. gambiae* mosquitoes after mating. We identify an atrial-specific *Mating-Induced Stimulator of Oogenesis* (*MISO*) that is regulated by and interacts with 20E. This interaction translates the male hormonal signal into an increased expression of a major vitellogenic lipid transporter, facilitating oocyte development via the accumulation of lipids in the ovaries.

## Results

### *MISO* Is a Mating-Dependent Regulator of Oogenesis

Our previous studies had identified a gene (*AGAP002620*, henceforth referred to as *MISO*) that is highly upregulated in the atrium during the first day after mating [49]. This gene encodes a glycine-rich protein of 152 aminoacids with no known functional domains. After confirming the atrium-specific, mating-induced expression of this gene (Figure S1A,B), we decided to examine whether *MISO* is involved in the regulation of two female postmating responses, oogenesis and oviposition. Consistent with a possible role in these processes, immunofluorescence and confocal microscopy analyses on virgin and mated atria at 12 and 24 h postmating (hpm) identified the protein in the ampullae, the tissues that connect the anterior part of the atrium to the oviducts (Figure S1C).

**Figure 1:**
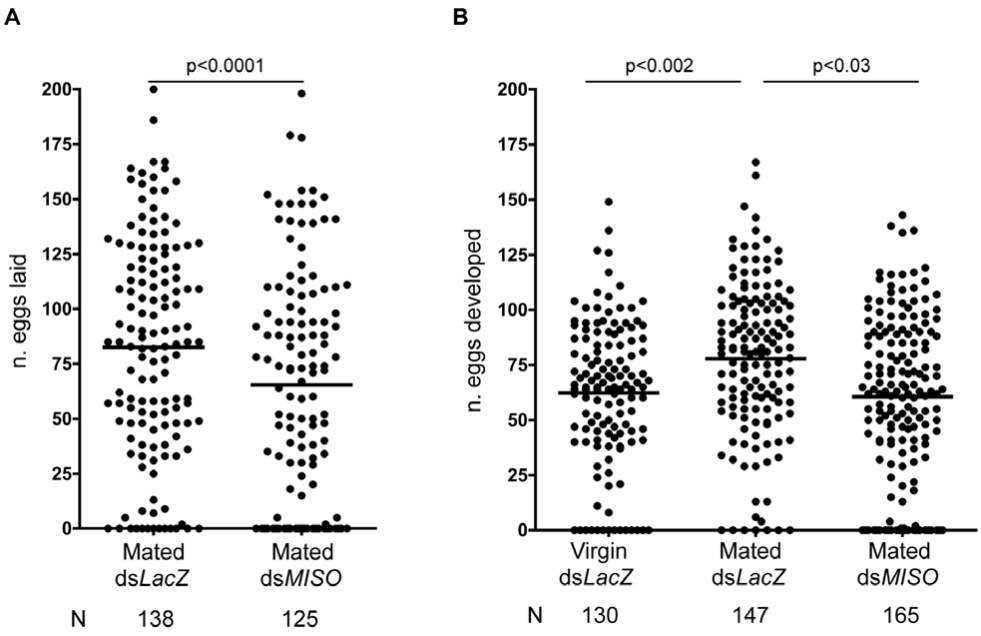
*MISO* knockdown decreases egg production to virgin levels. (A) Mated females injected with ds*RNA* were blood fed and allowed to lay eggs 3 d post-blood-feeding for 4 nights. Control females (ds*LacZ*) laid on average 82.5 eggs, while ds*MISO* oviposited a statistically significant lower number of eggs (65.4). The data are representative of three independent replicates. (B) Virgin or mated females injected with ds*RNA* were blood fed, and eggs developed inside the ovaries were counted 3 d post-blood-feeding without allowing oviposition. Mated ds*LacZ* produced on average 77.8 eggs, while virgin ds*LacZ* and mated ds*MISO* produced a statistically significant lower number of eggs (62.3 and 60.4, respectively). The data are representative of six independent replicates.

To study the function of *MISO*, we performed RNA interference (RNAi)–mediated gene silencing by injecting females with double-stranded RNAs (ds*RNAs*) targeting this gene (ds*MISO*) (transcript mean reduction = 74.4%±19.9%, one-sample *t* test: t_14_ = 14.45, *p*<0.0001, range 95%–31%; this knock-down completely abolished protein expression; Figure S1D,E). When injected females were mated, blood-fed, and allowed to lay eggs, a higher proportion of ds*MISO* females did not oviposit (29 out of 125, 23%) compared to control females injected with an unrelated control ds*RNA* (ds*LacZ*) (13 out of 138, 9%) (*χ* ^2^ = 9.281, *p* = 0.0023) (Table S1). Additionally, females injected with ds*MISO* laid a significantly smaller number of eggs (ds*LacZ*, 82.5 eggs; ds*MISO*, 65.4 eggs; Poisson regression, *χ* ^2^ = 236.6, *p*<0.0001) (Figure 1A). Dissection of the ovaries from both groups, however, revealed that this difference was due to a larger proportion of ds*MISO* females (16%) failing to develop eggs compared to controls (4%) (*χ* ^2^ = 11.68, *p* = 0.0006) (Table S1). The percentage of females with fully developed ovaries that did not oviposit was instead similar in both groups (ds*LacZ*, 6%; ds*MISO*, 9%; *χ* ^2^ = 0.5781, *p* = 0.4470), suggesting that MISO is important for egg development rather than for egg laying. No difference was detected in the fertility of the two groups (ds*LacZ*, 97%, *n* = 125; ds*MISO*, 96%, *n* = 96; Mann–Whitney test, U = 5089, *p* = 0 .3985) (unpublished data).

To further investigate the impact of *MISO* on oogenesis, ds*RNA*-injected females were mated and blood fed, and 3 d later, when oogenesis is normally completed, the ovaries of fully engorged females were dissected without allowing egg laying, and eggs were counted. Virgin ds*LacZ* females were included as controls to verify that under our experimental conditions virgins are less likely to produce eggs than mated females, as demonstrated by others [11]. Control females showed a 20% increase in egg production after mating (mated ds*LacZ*, 77.8 eggs; virgin ds*LacZ*, 62.3 eggs) (Figure 1B); however, this increase in egg production was completely abolished in mated ds*MISO* females (mated ds*MISO*, 60.4 eggs) (Figure 1B) (Poisson regression, *χ* ^2^ = 306.6, *p*<0.0001; Bonferroni multiple comparison post hoc test: virgin ds*LacZ* versus mated ds*LacZ*, *p* = 0.002; mated ds*LacZ* versus mated ds*MISO*, *p*<0.03; virgin ds*LacZ* versus mated ds*MISO*, *p*>0.05). Silencing of *MISO* before copulation therefore decreased egg development to levels observed in virgins, suggesting that this gene is required for the increase in oogenesis observed in *A. gambiae* females after mating.

### *MISO* Influences Lipid Accumulation in Developing Oocytes by Regulating the Expression of the Lipid Transporter *Lipophorin*

**Figure 2:**
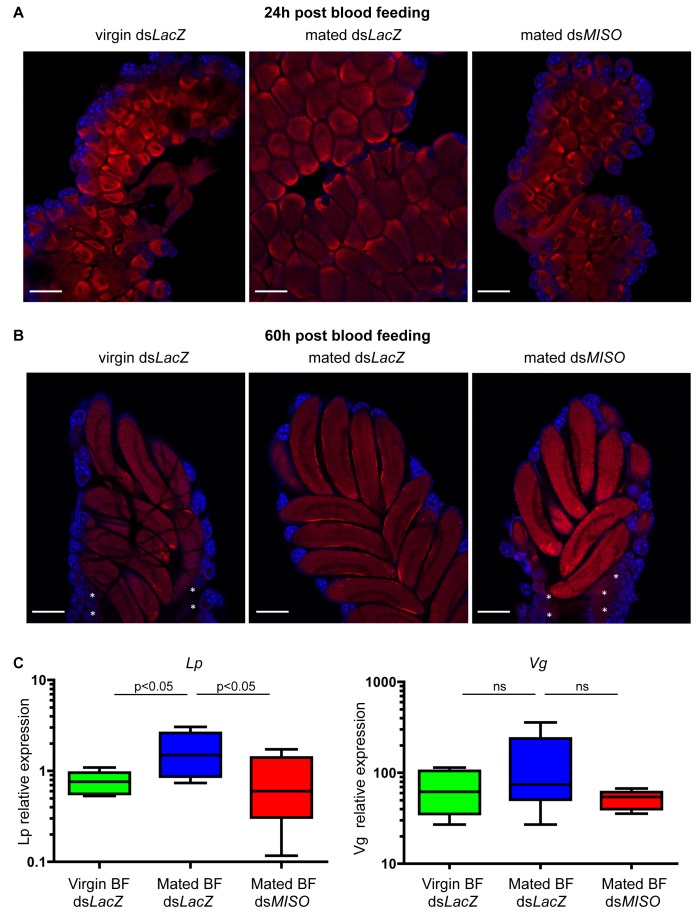
*MISO* silencing alters the expression of the lipid transporter *Lipophorin* in developing oocytes after blood feeding. (A and B) Immunofluorescence experiments on ovaries dissected from virgin and mated ds*LacZ* and mated ds*MISO* females stained with the lipid-binding reagent Nile-Red (red) at 24 h (A) and 60 h (B) post-blood-feeding. Asterisks in ds*MISO* and ds*LacZ* virgin ovaries indicate undeveloped primary follicles. Cell nuclei are labeled with DAPI (blue). Scale bar: 200 µm. (C) qRT-PCR of *Lp* and *Vg* from the fat body of virgin and mated ds*LacZ* and mated ds*MISO* females 24 h after blood feeding (BF). Expression levels (shown in logarithmic scale) were normalized to the housekeeping gene *RpL19*. The box-and-whisker diagrams represent five replicates of pools of 6–10 tissues.

After assessing the role of *MISO* in determining the increase in oogenesis induced by mating, we next analyzed the progression of oocyte development in mated ds*MISO* and control virgin and mated females at two time points (24 h and 60 h) after a blood meal. At 24 h postblood feeding, ds*MISO* follicles showed delayed development compared to mated ds*LacZ* controls, similar to what observed in the ovaries of virgin ds*LacZ* females (Figure 2A). By 60 h postblood feeding, oogenesis was completed in all three groups (Figure 2B); however, ds*MISO* (and virgin ds*LacZ*) ovaries showed a number of undeveloped primary follicles (indicated by asterisks in Figure 2B) in agreement with the finding that *MISO* silencing reduces egg development. A time course of five time points (12, 24, 36, 48, and 60 h) after blood feeding in virgin and mated females confirmed that, similar to virgin ds*LacZ* controls, mated ds*MISO* females exhibited a statistically significant delay in egg development, and only achieved oocytes of the size exhibited by mated ds*LacZ* individuals at 60 h postblood feeding (Figure S2 and Table S2). These results suggest that the effects of MISO on egg development are due to delayed or impaired accumulation of lipids into the growing oocytes.

The effects on oocyte growth observed in ds*MISO* females prompted us to analyze whether MISO plays a role in regulating the lipid transport to the oocyte. We therefore analyzed the expression levels of the vitellogenic lipid transporter *Lp* (*AGAP001826*) and the major YPP *Vg* (*AGAP004203*) in the fat body of blood-fed females at their peak of expression. In five different experiments, *Lp* transcript levels at 24 h after blood feeding were strongly reduced (54% mean reduction) in mated ds*MISO* females compared to mated controls, similar to virgin control levels (50% mean reduction) (Figure 2C) (Repeated Measures ANOVA, F_2,4_ = 8.142, *p* = 0.0118; Tukey's Multiple Comparison post hoc test: virgin ds*LacZ* versus mated ds*LacZ*, *p*<0.05; mated ds*LacZ* versus mated ds*MISO*, *p*<0.05; virgin ds*LacZ* versus mated ds*MISO*, *p*>0.05). *Vg* instead was not significantly affected by *MISO* silencing (Repeated Measures ANOVA, F_2,4_ = 1.362, *p* = 0.3098) (Figure 2C). Taken together, these results indicate that mating increases the blood feeding–induced expression of *Lp* and that this regulation is dependent on *MISO*.

### MISO Affects Male Ecdysteroid Titers in the Atrium and the Expression of 20E-Responsive Genes after Mating

**Figure 3:**
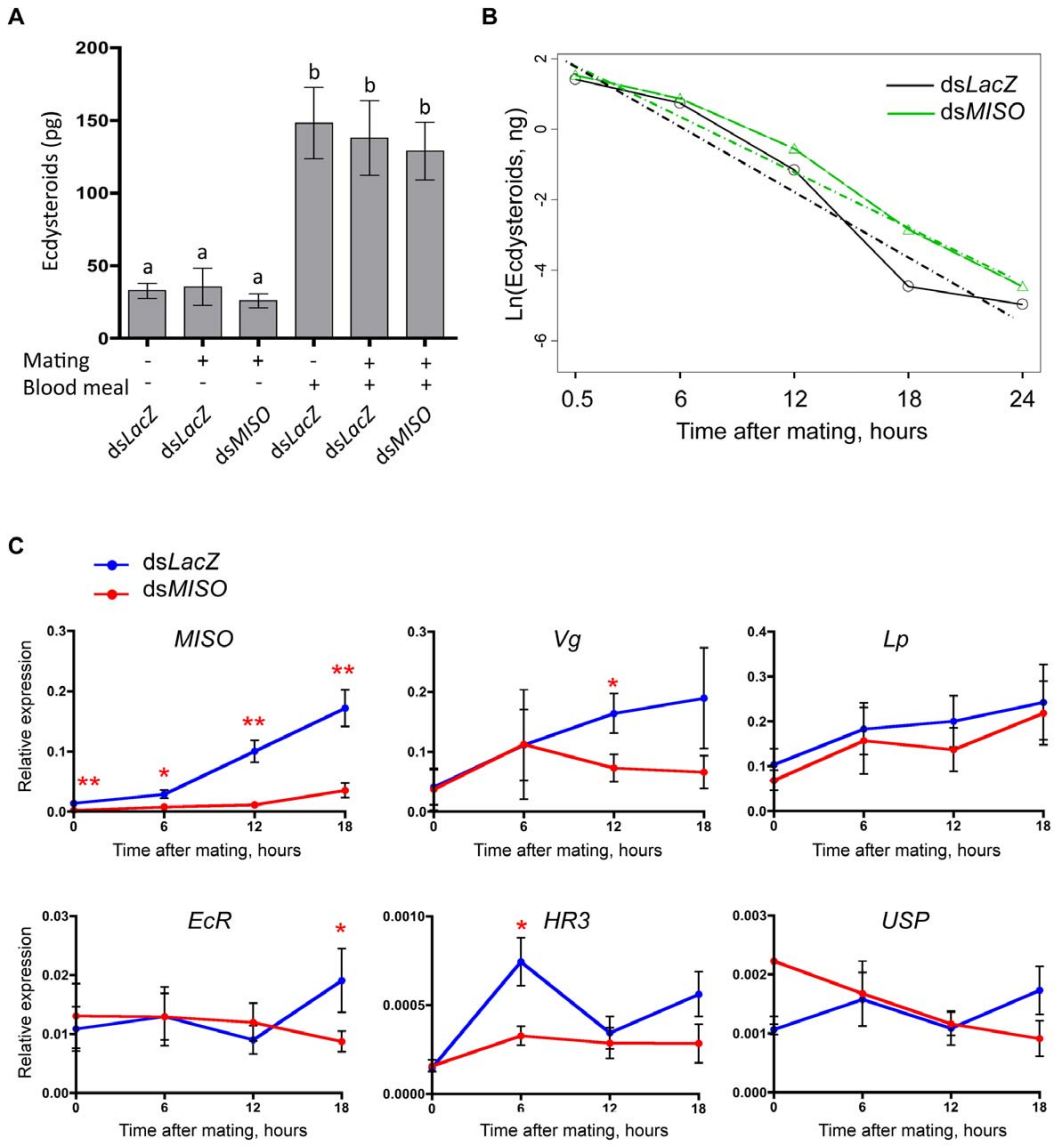
*MISO* silencing affects atrial 20E titers and reduces the activation of 20E-responsive genes after mating. (A) *In vitro* ovarian ecdysteroid secretion before and 18 h after a blood meal in virgin and mated ds*LacZ* and mated ds*MISO*. Graph shows data from eight individual ovaries. Data are represented as mean ± SEM. Means with the same letter are not significantly different (*p*>0.05). (B) Changes over time in the geometric mean of the ecdysteroid titer (natural logarithm) of ds*LacZ* (black solid line with circles) and ds*MISO* (green dashed line with triangles) females at 5 time points after mating (0.5, 6, 12, 18, and 24 hpm) with the mean trajectories estimated in regression mixed models (dashed and dotted lines). Nine replicates were performed using a pool of three atria each. (C) qRT-PCR of 5 20E-responsive genes (*Vg*, *Lp*, *EcR*, *HR3*, and *USP*) in ds*MISO* and ds*LacZ* females at different time points (0, 6, 12, 18 h) after mating. The levels of *MISO* after ds*MISO* injections are also shown. Four independent replicates were performed using a pool of 5–10 female abdomens. Expression was normalized to the housekeeping gene *RpL19*. Data are represented as mean ± SEM. One or two asterisks represent *p*<0.05 and *p*<0.001, respectively.

In *A. aegypti* mosquitoes, the expression of the *Vg* and *Lp* after blood feeding is induced by the function of 20E produced by the female [29],[30], and a similar regulation occurs also in *A. gambiae* [22]. We tested whether the MISO-mediated upregulation in the expression of *Lp* in mated females after a blood meal was caused by an increased production of this hormone. We measured ecdysteroid levels secreted *in vitro* by the ovaries of virgin and mated ds*LacZ* and mated ds*MISO* females before and 18 h after a blood meal, at their peak of secretion [48]. As expected, blood feeding strongly increased the steroidogenic capacity of the ovaries (Figure 3A) (one-way ANOVA, F_5,42_ = 11.17, *p*<0.0001; post hoc Tukey's multiple comparison, non–blood-fed versus blood-fed groups, *p*<0.01). However, no differences between virgin and mated females were observed, and silencing of *MISO* did not affect ecdysteroid secretion levels (*p*>0.05) (Figure 3A).

Besides being produced by the female after blood feeding, in *A. gambiae* 20E is also synthesized in the MAGs and transferred to females during mating [48]. We therefore hypothesized that sexually transferred 20E may play a role in the MISO-mediated regulation of female physiology after mating. As a first step, we determined that the MAG-produced 20E is transferred to the female as part of the mating plug (Figure S3A). By 12 hpm, 20E localization was restricted to the anterior portion of the plug that is enclosed within the ampullae (Figure S3A), where MISO also localizes (Figure S1C). The amount of 20E detected in the MAGs corresponded to a mean of 632 pg (±17 pg), consistent with previous findings by others (Figure S3B) [48]. Interestingly, no 20E could be detected in the male reproductive tissues of two mosquito species, *Anopheles albimanus* and *A. aegypti*, which do not produce mating plugs (Figure S3B).

We next investigated whether MISO affects the activity of 20E transferred by males during copulation. To this aim, we analyzed steroid hormone levels in the atria of ds*LacZ* and ds*MISO* females at five time points after mating (0.5, 6, 12, 18, and 24 hpm) to monitor 20E release from the mating plug over time. Immediately after mating (0.5 hpm), the atria of control and ds*MISO* females contained similar hormone titers (Figure 3B). Ecdysteroid levels in the atria of controls were statistically significantly decreased at the four later time points (Wilcoxon test, *p*<0.001) and reached about 3 pg per individual by 24 hpm, suggesting that 1 d after copulation the steroids have been fully released from the mating plug and have circulated out of the atrium. Interestingly, ecdysteroid titers declined more slowly in the atria of ds*MISO* females (P-mixed effects model, *p* = 0.055) (Figure 3B). No 20E was detected in the atria of virgin females (unpublished data), confirming that this hormone in the female is only produced after blood feeding. These results suggest that silencing of *MISO* impairs the release of ecdysteroids from the plug and/or their diffusion from the atrium, possibly affecting their function.

To confirm the latter hypothesis, we analyzed the transcription levels of five 20E-responsive genes at three time points after mating (6, 12, and 18 hpm) in the two RNAi-injected groups. If MISO impairs the release of 20E from the atrium, then the expression levels of these genes in surrounding tissues should be altered in ds*MISO* females compared to controls. Besides *Vg* and *Lp* [28],[29], we analyzed *Ecdysone Receptor* (*EcR*, *AGAP012211*) [31], *Ultraspiracle* (*USP*, *AGAP002095*) [50],[51], and *Hormone Receptor 3* (*HR3*, *AGAP009002*) [52]. As mentioned above, *EcR* is a nuclear receptor that in conjunction with *USP* activates transcription of downstream genes upon binding of 20E [31],[50],[51], while HR3 is known to interact directly with EcR [52]. Three genes exhibited a significant reduction in postmating expression in ds*MISO* females over the time frame analyzed: *HR3* was downregulated by 50% at 6 hpm (*t* test, t_6_ = 2.431, *p* = 0.0256), *Vg* was reduced by 54% at 12 hpm (*t* test, t_6_ = 2.785, *p* = 0.0159), while *EcR* was decreased by 44% at 18 hpm (*t* test, t_6_ = 1.876, *p* = 0.0587) (Figure 3C). The expression levels of *Lp* and *USP* did not significantly differ between control and experimental females (Figure 3C).

All together, these results show that *MISO* silencing impairs both the titers of 20E in the atrium and the expression of 20E-responsive genes after mating, reinforcing the hypothesis that MISO influences the function of male-derived ecdysteroids delivered by the mating plug.

### MISO Interacts with and Is Regulated by Male-Transferred 20E

**Figure 4:**
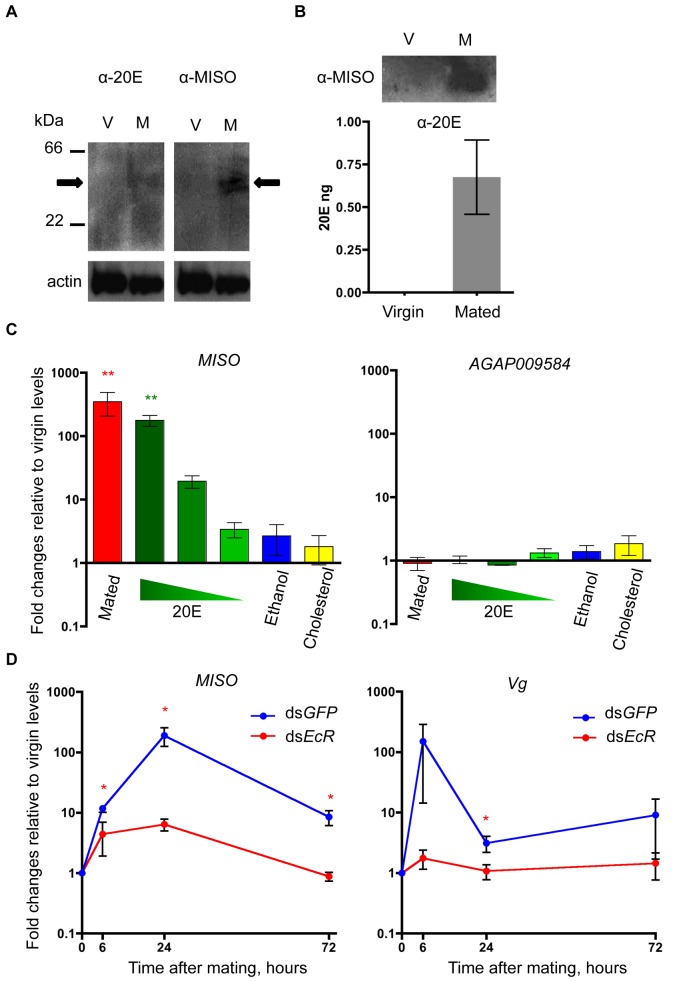
MISO is induced by and interacts with 20E in the female atrium. (A) Western blot under native nondenaturing gel condition using anti-20E and anti-MISO antibodies on atria of virgin or mated (8 hpm) females. Left and right arrowheads indicate 20E and MISO positive bands, respectively. (B) Co-immunoprecipitation of MISO and 20E in atria of virgin or mated (8 hpm) females. The anti-MISO immunoprecipitation (IP) was quantified with anti-20E ELISA. Mated atria showed a mean of 0.68 ng, while in virgin atria no 20E was detected. Three experiments were performed using a pool of 50 atria each, one-third used for the IP (upper panel) and two-thirds for the ELISA (lower panel). Data are represented as mean ± SEM. (C) *MISO* expression in atria dissected from females previously injected in the hemolymph with three 10-fold dilutions of 20E in ethanol (10% v/v) (starting from 2.5 µg of 20E per mosquito). Ethanol (10% v/v) and water-soluble cholesterol (0.25 µg/mosquito) were used as controls. Expression levels were measured 24 h after injection or mating, and were normalized to *RpL19* and then to virgin levels in each replicate. A minimum of three replicates were performed for each condition. Data are represented as mean ± SEM. (D) qRT-PCR of *MISO* and *Vg* in females injected with ds*EcR*, analyzed at 6, 24, and 72 hpm. Expression levels (shown in logarithmic scale as fold changes relative to age-matched virgins) were normalized to the housekeeping gene *RpL19*. The analysis was performed in four replicates on pools of 5–10 female lower reproductive tracts. Data are represented as mean ± SEM. The asterisk indicates *p*<0.05.

We next investigated whether the effects of *MISO* silencing on 20E titers and on the expression of 20E-responsive genes were caused by a possible interaction between MISO and 20E. To this aim, Western blot analyses were performed under native (i.e., nondenaturing) conditions. An anti-20E antibody detected a band of approximately 40 kDa in the atria of mated female (8 hpm) that was not detected in virgin extracts (Figure 4A). This band reacted also with anti-MISO antibodies, suggesting that the two factors are part of the same complex (Figure 4A). Moreover, immunoprecipitation of MISO in extracts of virgin and mated atria at 8 hpm followed by an ELISA coupled with anti-20E antibodies detected significant amounts of 20E co-immunoprecipitating in mated females, while no signal was observed in virgins (Figure 4B). All together these results suggest an interaction between MISO and 20E in the atrium of females after mating.

As 20E is known to regulate the expression of genes that are ultimately responsible for its function (reviewed in [53]), we next analyzed whether this steroid hormone plays a role in the expression of *MISO* in the atrium. To this aim, we injected three 10-fold dilutions of 20E into the hemolymph of virgin females, and analyzed *MISO* transcript levels specifically in the atrium (where the gene is not normally expressed in virgin females) at 24 h postinjection. At the highest concentration, 20E significantly induced *MISO* expression to levels similar to those achieved by mating (178- and 349-fold induction, respectively) (one-way ANOVA, F_6,23_ = 14.79, *p*<0.0001; post hoc Dunnett's multiple comparison against virgins, *p*<0.01), while the ethanol and cholesterol controls had no effect (Figure 4C). At lower dilutions, 20E injections increased *MISO* expression levels relative to controls, however this effect was not statistically significant. No effect on *MISO* expression was seen in tissues other than the atrium, confirming the tissue-specific restriction of expression of this gene (unpublished data). The expression of *AGAP009584*, an atrial gene that is not modulated by mating [10],[49], was not induced by the injection of any of the 20E dilutions (Figure 4C) (one-way ANOVA, F_6,23_ = 0.5089, *p* = 0.7947). Only the highest concentration of injected 20E achieved physiological atrial concentrations similar to those transferred during mating (Figure S4), explaining the observed titration-dependent upregulation of *MISO* expression.

Finally, to further confirm that 20E induces *MISO* expression in the atrium, we tested MISO induction levels in the absence of the 20E receptor EcR. We injected virgin females with ds*RNA* targeting *EcR*, and analyzed levels of *MISO* induction after mating. In four different experiments, injection of ds*EcR* (transcript mean reduction = 45%; one-sample *t* test, t_3 = _7.069, *p* = 0.0058, range 63%–36%) impaired *MISO* induction at 24 hpm by an average of 30-fold compared to injected controls (*t* test, t_6_ = 2.466, *p* = 0.0244) (Figure 4D), reinforcing the notion that the expression of this gene after mating is regulated by male-transferred 20E. Interestingly, *EcR* silencing also reduced transcript levels of *Vg* (24 hpm: *t* test, t_6_ = 2.106, *p* = 0.0399), as expected as this gene is under the control of 20E and its expression is induced by both blood feeding and mating in *A. gambiae* (Figure 4D) [22],[49]. These data demonstrate that the mating-induced expression of *MISO* is under the control of sexually transferred 20E, and that *EcR* mediates this regulation.

## Discussion

In this study we unravel a major male–female molecular interaction that switches females to a mated state in terms of egg development and modulates their postmating physiology. We identify a female atrial protein, MISO, which is responsible for the increase in egg production after mating. Silencing of *MISO* reverts fecundity of mated females back to virgin levels, completely abolishing the effects of mating on oogenesis (Figure 1). Moreover we demonstrate that MISO is induced by and interacts with the steroid hormone 20E transferred by the male (Figure 4). Sexually transferred 20E therefore acts as a “mating signal” that regulates female postmating physiology, and its interaction with MISO translates this signal into increased oogenesis in blood-fed females. To our knowledge, this is the first demonstration of an interaction between a male allohormone and a female protein in insects. The identification of this novel interaction in *A. gambiae* expands our knowledge of male–female molecular partnerships important for reproductive success, to date limited to few examples from *Drosophila* (reviewed in [54]).

The mating-induced increase in egg development seen in our experimental settings only partially reflects the deep impact that mating has on oogenesis in field conditions. Blood-fed virgins from natural mosquito populations rarely develop eggs after a single blood meal [14]–[16], presumably because of limited nutritional reserves from larval stages [17]. MISO may therefore represent a mating sensor that directs precious resources towards oogenesis only when females are inseminated. Indeed in two different phenotypic assays, MISO influenced pregravid behavior, and similar to virgin females, approximately 15% of ds*MISO* mated females completely failed to develop eggs compared to 4% of mated controls (Figure 1 and Table S1). It is reasonable to speculate that this effect would be much more pronounced in conditions of limiting resources such as those possibly available in field settings.

The interaction between MISO and 20E affects the function of the steroid hormone, as demonstrated by the effects of *MISO* silencing on 20E titers in the atrium and on the expression of a number of 20E-responsive genes (Figure 3B,C). Although the protein does not have any known functional domains that suggest a role as a sterol carrier, our data indicate that MISO facilitates the release of 20E from the mating plug and its diffusion from the atrium (Figure 3B). Further studies may help elucidating the mechanism by which this female atrial protein regulates 20E function. On the other hand, the finding that sexually transferred 20E induces the atrial-specific expression of *MISO* via the *EcR* receptor shows a remarkable mutual cooperation between the two factors (Figure 4C,D). Preventing males from producing and transferring 20E will clarify the full extent of the role that this ecdysteroid plays in regulating female postmating physiology and behavior.

A number of hypotheses can be formulated on the downstream events triggered by the interaction of MISO and 20E that lead to increased fecundity. One possibility is that this interaction may prime the fat body to respond to the female-derived ecdysteroids synthesized after a blood meal. This hypothesis is strengthened by the observations that mated ds*MISO* females experienced a reduced induction in *Lp* expression after blood feeding compared to controls, paralleled by delayed or impaired oocyte growth (Figure 2, Figure S2, and Table S2). The higher level of *Lp* expression seen in control mated females is not due to an increased release of ecdysteroids from the ovaries after blood feeding, as ecdysteroid titers were similar in control and ds*MISO* females (Figure 3A). Interestingly, *MISO* silencing affects the expression of *Vg* and *Lp* differentially: while the most prominent effect on *Lp* occurs after blood feeding (Figure 2C), *Vg* transcript levels are repressed only after mating (Figure 3C). This observation suggests a bimodal role for the MISO–20E interaction: a local effect on the expression of mating-responsive genes such as *Vg* that may regulate the function of reproductive tissues and possibly the remodeling of atrial cells observed after mating [49], and a later effect due to 20E release from the atrium that may control the response of the fat body to blood feeding, thereby affecting *Lp* transcript levels and egg development. Importantly, these results are consistent with a recent report that identified Lp rather than Vg as the factor most relevant for egg development in *A. gambiae* [55]. Another possible mechanism is that sexually transferred 20E may regulate resorption of ovarian follicles. In *A. aegypti* the interplay between JH and 20E influences the fate of follicular resorption during the previtellogenic and vitellogenic stages [56]. Low JH titers during the previtellogenic stage result in higher follicular resorption that can be prevented by the application of methoprene, a JH mimic [57]. 20E can also stimulate resorption of “poor quality” follicles that express low levels of Vg and Lp receptors [58], probably by a caspase-mediated cell death mechanism [59]. In *A. gambiae* male transferred 20E may therefore act cooperatively with female-derived JH in determining correct follicular resorption. Alternatively, the large amount of 20E transferred from the MAGs, that as confirmed here exceeds the concentration produced by the ovaries after blood feeding [48], may increase the number of developing oocytes by causing yolk accumulation in secondary follicles already during the first blood meal. This process has been observed in *A. aegypti* [60] and *A. stephensi* [61] after 20E injection.

Mating does not modulate egg development in all anopheline species. For instance, oogenesis is not affected by copulation in the central American malaria vector *A. albimanus* [62], and interestingly, we could not detect any 20E in the MAGs of this mosquito species (Figure S3B). This result suggests that the effect of mating on fecundity in anophelines might be directly linked to the presence of 20E in the male reproductive tract. Intriguingly, secretion of lower 20E titers in *A. gambiae* compared to *A. albimanus* females after a blood meal [22],[63] may be due to the availability of 20E from males in the former species. An increase in egg development following mating is also seen in *A. aegypti* [39], however the absence of 20E in the MAGs of this species suggests that this effect is caused by a different mechanism (Figure S3B). This increase may be regulated by MAG proteins stimulating the synthesis of growth hormones, as in the case of the stimulation of JH synthesis by Sex Peptide in *Drosophila* [33]. Indeed the existence of a Sex Peptide–like factor inducing postcopulatory changes in *A. aegypti* is supported by the observation that MAG extracts injected into virgin females trigger oviposition after blood feeding [64],[65], contrary to *A. gambiae* where they have no effect [13]. Alternatively, hormones other than 20E produced by the male and transferred during mating may play this role. JH has been detected in the MAGs of *A. aegypti* [66], and the application of the JH analog methoprene to virgin *A. aegypti* females enhances oogenesis [39]. No evidence of JH synthesis exists in the MAGs of *A. gambiae*, and unlike *A. aegypti*, application of methoprene to blood-fed females inhibits egg maturation and vitellogenesis [22], suggesting differences in the mechanism of oogenesis in the two species. The analysis of the synthesis of 20E in the MAGs of other mosquito species, facilitated by the sequencing of an additional 16 anopheline genomes (http://www.vectorbase.com), will clarify the existence of a possible correlation between mating plug formation and 20E synthesis in the male, two reproductive features that are both present in *A. gambiae* but not in *A. albimanus* and *A. aegypti*, and between the sexual transfer of 20E and the occurrence of mating-induced oogenesis.

Finally, the identification of a previously uncharacterized reproductive pathway in *A. gambiae* has promise for the development of tools for the control of malaria-transmitting mosquito populations. The effects of the 20E-MISO partnership are likely to be more prominent in field mosquitoes, where nutritional resources are limited and egg development rarely occurs in virgins. Manipulation of this interaction with specific inhibitors or with genetically manipulated males impaired in 20E synthesis might therefore offer an attractive option for reducing the reproductive output of natural *Anopheles* populations. Moreover, interfering with the mating-induced pathway of oogenesis may have an effect on the development of *Plasmodium* malaria parasites. A recent study has shown that the expression of *Vg* and *Lp* reduces the mosquito *Plasmodium*-killing efficiency mediated by TEP-1, the principal antiparasitic factor in *A. gambiae* [55]. As YPPs are regulated after a blood meal via a MISO-dependent mechanism, the 20E–MISO interaction may play a role in the modulation of *Plasmodium* development in *A. gambiae*.

## Materials and Methods

### Mosquito Procedures

Mosquitoes from a laboratory colony of the *A. gambiae* G3 strain were reared under standard conditions [26–28°C, 65%–80% relative humidity, 12 h∶12 h Light/Darkness (L∶D) photoperiod]. For mating experiments, mosquitoes were separated by sex as pupae and raised in cages supplied with sucrose *ad libitum*. Matings were performed as described previously, and couples were captured *in copula* [49].

### RNA Interference

A 397 bp region corresponding to the coding sequence of *MISO* (*AGAP002620*) was amplified from atrial cDNA 24 hpm using specific primers FWD: 5′GGTGTTGCCATTGTGTGTGT-3′ and REV: 5′AGTACTCGGCCAGCTGAATG -3′ and cloned into the pLL10 plasmid [67]. A 435 bp region corresponding to *AGAP012211* (*EcR*) was amplified from female abdomen cDNA using the primers FWD: 5′CTGCTCCAGTGAGGTGATGA-3′ and REV: 5′GGCAGCTTACGGTTCTTCAG-3′, while a 495 bp portion of the eGFP control gene was amplified using the primers FWD: 5′TGTTCTGCTGGTAGTGGTCG-3′ and REV: 5′ACGTAAACGGCCACAAGTTC-3′; both amplicons were cloned into pCR2.1 (Invitrogen). These constructs were then used to synthetize dsRNAs targeting the different genes, following established protocols [10],[67],[68]. Females were sexed as pupae and injected with 69 nl of dsRNA (4 mg/ml) within 24 h of eclosion. Surviving females were allowed to mate with 4-d-old virgin males 3 d after injection. Mated females were then used for phenotypic assays or dissected for qRT-PCR analysis. RNA extraction, cDNA synthesis, and SYBR-green based qRT-PCR were performed as described previously [49] using the primers listed in Table S3. The ribosomal protein gene *RpL19* (*AGAP004422*) was used for normalization, using previously described primers [49].

### Oviposition, Egg Development, and Fertility Assays

Three days after ds*RNA* injections, females were captured during mating and kept in isolation until blood feeding. Females were blood fed *ab libitum* on human blood. Partially fed or unfed mosquitoes were removed. For oviposition and fertility assays, 3 d after the blood meal, females were put into individual oviposition cups for 4 nights. After completion of oviposition, eggs were counted under the microscope and those that hatched into a larva were scored as fertile. For the egg development assay, abdomens were dissected 3 d after blood feeding, and eggs developed inside the ovaries were counted under the microscope.

### Polyclonal Anti-MISO Antibodies

Affinity-purified polyclonal antibodies against MISO were raised in rabbit against the peptide epitope CSNGPSSSYGPPRNT by a commercial supplier (GenScript Corp., Piscataway, NJ).

### Immunoblots

Female tissues were homogenized in 20 µl RIPA buffer (10 mM Tris/HCl pH 7.6, 100 mM NaCl, 10 mM EDTA, 0.5%, Nonidet P40, 0.5% Triton ×100, 1× proteases inhibitor from Roche). Samples were centrifuged at 13,000 rpm for 15 min at 4°C. The supernatant was diluted into NuPAGE reducing agent and sample buffer (Invitrogen), heated at 70°C for 10 min, and applied to precast NuPAGE gels (Invitrogen) under reducing conditions according to the manufacturer's instructions. For native conditions, protein extraction was performed by homogenizing the tissues in a hypotonic solution (10 mM Tris/HCl pH 7.6, 10 mM NaCl, 10 mM EDTA, 1× protease inhibitor from Roche) followed by centrifugation at 13,000 rpm for 15 min at 4°C. The soluble phase was then loaded onto an acrylamide gel in the absence of SDS. Proteins were transferred to a Hybond ECL membrane using the XCell II Blot module (Invitrogen). Membranes were immunostained using standard protocols with the following primary antibody titres: anti-MISO, 0.96 mg/ml; anti-20E (1∶10 dilution, Cayman Chemicals); and anti-β-actin (1∶1,000 dilution, Santa Cruz Biotechnologies). HRP-conjugated secondary antibodies (Santa Cruz Biotechnologies) were used at a dilution of 1∶10,000. Bands were visualized using ECL Western blotting detection reagents (GE Healthcare). Reprobing with additional primary antibodies was performed after incubating membranes in stripping solution (10 mM Tris/HCl PH 6.8, 100 mM DTT, SDS 2%) at 50°C for 30 min. Before adding the new primary antibody, incubation with the secondary antibody used in the first analysis was tested by ECL to exclude any signal from the previous incubation.

### Immunofluorescence and Confocal Analysis

MAGs or female reproductive tracts from 3–4-d-old mosquitoes (virgins and mated) were dissected on ice, fixed in 4% formaldehyde, washed in PBS, then blocked and permeabilized in PBS with 1% BSA and 0.1% saponin. Samples were incubated in either 3 mg/ml anti-MISO or a 1∶10 dilution anti-20E (Cayman Chemicals), then a 1∶1,000 dilution of anti-rabbit Alexa-Fluor 488 (Invitrogen). Alternatively, ovaries were stained with 1∶1,000 dilution of Nile-Red (10 mg/ml in DMSO, Sigma-Aldrich). Tissues were then mounted in DAPI-containing Vectashield medium (Vector Laboratories, Inc.) and visualized using a Point Scanning Confocal microscope Nikon TE2000 or a Zeiss Axio Observer inverted fluorescent microscope with apotome.

### *In Vitro* Ovarian Culture

Ovaries of dsRNA-injected females were dissected from virgin and mated mosquitoes before or after 18 h after a blood meal. Blood feeding was performed 1 h after mating. Ovaries of mated non-blood-fed females were dissected 19 h after copulation. After dissection in Schneider medium (Sigma-Aldrich), individual pairs of ovaries were separately transferred to 50 µl of Schneider medium and incubated for 5 h at 25°C. After incubation, culture medium was stored at −80°C until ecdysteroid quantification.

### 20E ELISA

Atria from groups of three virgin females or from groups of three mated females at different time points after mating, previously injected with ds*MISO* or ds*LacZ*, were placed in 50 µl methanol and frozen at −80°C. Alternatively, MAGs or testes from 10 *A. gambiae*, *A. albimanus*, and *A. aegypti* males were dissected and placed in 50 µl methanol. Tissues were then homogenized and loaded into separate wells of a 96-well plate pre-coated with mouse anti-rabbit IgG (Cayman Chemical). For the analysis of the *in vitro* ovarian ecdysteroid secretion, 50 µl of Schneider medium where the ovaries have been incubated were directly loaded into the gel. A standard curve was prepared from 18 ng 20E (Sigma-Aldrich) in methanol or Schneider medium (Sigma), with a series of seven 3-fold dilutions. After evaporation of the methanol, 50 µl of each of the following solutions were added: Enzyme ImmunoAssay Buffer (0.1 M phosphate solution containing 0.1% BSA, 0.4 M sodium chloride, 1 mM EDTA, and 0.01% sodium azide); 20E acetylcholinesterase (AChE) Tracer, which is a covalent conjugate of 20E and AChE; and anti-20E rabbit IgG (Cayman Chemical). The plate was incubated with the solutions overnight at 4°C, washed with PBS 1× containing 0.05% TWEEN20, incubated with 200 µl Ellmans reagent (5,5′-dithiobis-(2-nitrobenzoic acid)) (Cayman Chemical), and finally developed for 90–120 min and measured in an ELISA reader at 420 nm.

### 20E Injections

Three-day-old females were injected with different quantities (2.5 µg, 0.25 µg, and 0.025 µg) of 20E (138 nl of 10% ethanol solution). As controls, either the same volume of 10% ethanol or 0.25 µg of water-soluble cholesterol (which is the maximum soluble concentration) (Sigma-Aldrich) were injected. Female lower reproductive tracts (LRT, atrium, spermatheca, and parovarium) were dissected 24 h after injection and analyzed by qRT-PCR. Three replicates were performed containing 6–8 tissues per replicate. LRTs were also dissected and analyzed by qPCR from noninjected virgin females and from mated females at 24 hpm.

### Immunoprecipitation Experiments

Fifty atria from virgin and mated (8 hpm) females were dissected and homogenized in 15 µl of hypotonic solution (10 mM Tris/HCl pH 7.6, 10 mM NaCl, 10 mM EDTA, 1× protease inhibitor from Roche) and centrifuged at 13,000 rpm for 15 min at 4°C. The soluble phase was then incubated for 1 h at 4°C under gentle rocking with 2 mg of anti-MISO rabbit IgG that had been previously linked to Dynabeads protein A (Invitrogen) in a 10 min incubation at 25°C under gentle rocking followed by three PBS 1× washes. The immunoprecipitate was washed three times with PBS 1× and split in two aliquots: one-third of the total volume was utilized in a Western blot incubated with anti-MISO, while the remaining two-thirds were diluted with 100 µl of methanol, to extract 20E, and kept at −80°C. The methanol solution was then analyzed with an anti-20E ELISA. As controls, 25 ng of 20E were incubated under the same conditions with 2 mg of Rabbit anti-MISO linked to Dynabeads protein A to measure the unspecific binding of 20E to the antibody or to the Dynabeads. All samples were also immunoprecipitated using pre-immune rabbit IgG to control for unspecific bindings. ELISA quantification was performed normalizing the signal to anti-MISO rabbit IgG-Dynabeads protein A incubated in methanol.

### Statistical Analysis

To examine the effects of MISO on oviposition and egg development, we utilized a generalized linear model approach where the number of eggs was modeled with a log link function and Poisson distribution function using SAS Proc GenMod (SAS, Inc., Cary, NC). Replicate was also included as a covariate in each of these analyses. Post hoc comparisons for fecundity were made using the Bonferroni Multiple Comparison Procedure in SAS (SAS, Inc.).

Differences in the number of females that fail to lay or to develop eggs (Table S1) between different groups were analyzed with a chi-square test using Prism 5.0 (GraphPad Software, Inc., La Jolla, CA). To test for difference in gene expression between two or more treatments (Figure 2C, Figure 3C, Figure 4), we used *t* test or ANOVA test, respectively, using Prism (GraphPad Software, Inc). Similarly, ecdysteroid secretion by ovaries and oocyte lengths between different groups were compared using ANOVA test. Differences in fertility between ds*LacZ* and ds*MISO* were examined through Mann–Whitney (Prism, GraphPad Software, Inc.).

For 20E titers in mated atria (Figure 3B), a Wilcoxon test was used to compare the natural logarithm transformed ecdysteroid levels of each group at different time points. Furthermore, we compared trajectories of steroid hormone levels of ds*MISO* and ds*LacZ* female groups through a mixed model, with natural logarithm transformed steroid levels and a random intercept to accommodate within female correlations measured at the five time points after mating (0.5, 6, 12, 18, and 24 hpm). Since we did not expect to find any differences in the mean levels of steroid at the first time point, we forced a common intercept for *dsLacZ* and ds*MISO* females by including in these models only a fixed effect for time. Statistical significance in the trajectory of the geometric mean of steroid levels (exp {mean[log(steroid)]}) between the two female groups was tested through an interaction term between time and female group (S.plus 8.0, TIBCO Software).

## Supporting Information

Figure S1 ***MISO*** **is strongly induced in the atrium after mating and is secreted in the ampullae.** (A) Quantitative RT-PCR (qRT-PCR) showing *MISO* expression in three conditions: virgin females after a blood meal (VBf), mated females (M), and mated females that have been blood fed immediately after mating (MBf). Atria, ovaries, and the rest of the body (carcass) were analyzed at different days (1, 3, and 6 d) postmating and/or blood feeding, and in age-matched virgin females. Expression levels (shown in logarithmic scale) were normalized to the housekeeping gene *RpL19*. The analysis was performed in three replicates on pools of 5–10 tissues, and data are represented as mean ± SEM. (B) Immunoblot analysis of MISO using a polyclonal antibody raised against a peptide fragment of the protein. Atria were dissected from different groups of females: virgins (V); mated (M) at 24 hpm; virgin blood fed (VBf) dissected at 24 h post-blood-feeding; mated blood fed (MBf), dissected at 24 h postmating and blood feeding; and MBf dissected after egg laying (EL). Immunoreactive bands (arrow) corresponding to the predicted 15 kDa size of MISO were detected in M, MBf, and EL atria. Actin was used as loading control. (C) Confocal analysis of MISO (green) in the atrium of virgin and mated females. The images next to the bright field (BF, scale bar: 100 µm) are magnifications (xy section, scale bar: 50 µm) of the regions indicated in the inset. At 12 hpm the mating plug is visible in the atrium (arrowhead). Cell nuclei (blue) are labeled with DAPI. (D) cDNAs from 15 independent replicates of ds*MISO* injections in virgin females analyzed by qRT-PCR at 24 hpm. *RpL19* relative expression levels were compared between ds*MISO-* and ds*LacZ*-injected females (dotted line). Data are represented as a box-and-whisker diagram. (E) Immunoblot analysis of the efficacy of *MISO* silencing in protein extracts from atria, ovaries, and eggs. Atria and ovaries were dissected from virgin or mated females at 24 hpm that were injected with either ds*MISO* or ds*LacZ*, as indicated. Eggs were collected 1–4 h after oviposition. Actin was used as loading control. The arrow indicates the expected size for MISO.
(TIF)

Figure S2 ***MISO*** **silencing induces a delay in ovarian development.** Immunofluorescence of oocyte development in ovaries dissected from ds*MISO* or ds*LacZ*-injected virgin or mated females at five points (12, 24, 36, 48, and 60 h) after blood feeding. Nile-Red (red) and DAPI (blue) were used to stain lipids and cell nuclei, respectively. Scale bar: 50 µm.
(TIF)

Figure S3 **20E localization in MAGs and atrium and quantification in male reproductive tracts from three mosquito species.** (A) MAGs dissected from virgin males (MAGs) and atria dissected from virgin (V) and mated females at two time points after mating (0.5 hpm and 12 hpm) were dissected and incubated with anti-20E antibody (green). Cell nuclei (blue) are labeled with DAPI. Scale bar of the bright field (BF): 100 µm. The images next to the bright field (BF) are a magnification (xy section) of the region indicated by the inset (scale bar: 50 µm). (B) ELISA quantifications of 20E levels in MAGs and testes from either *A. gambiae*, *A. albimanus*, or *A. aegypti* males. A pool of 10 tissues was used for each of three replicates. Data are represented as mean ± SEM.
(TIF)

Figure S4 **20E quantification in the atrium after injection.** ELISA quantification of 20E levels in female atria was performed prior or post injection (at 0.5 h, 6 h, and 24 h postinjection) of different 20E dilutions in the hemolymph of virgin females, or at the same time points after mating. Three 1∶10 dilutions starting from 2.5 µg per mosquito were injected. Ethanol injections were used as a control. A pool of 10 atria was used for each of three replicates. Data are represented as mean ± SEM.
(TIF)

Table S1 **Summary of phenotypic analysis of ds** ***MISO*** **-injected females.** *MISO* knockdown results in higher proportion of females that fail to develop eggs in both the oviposition and the egg development (oogenesis) assay (ds*LacZ* mated versus ds*MISO* mated: *χ* ^2^ = 6.864, *p* = 0.0088; ds*LacZ* mated versus ds*LacZ* virgin: *χ* ^2^ = 3.553, *p* = 0.0594). Among females that completed oogenesis, injections of ds*MISO* reduced the number of developed eggs (oviposition: *t* test: t_219_ = 0.9994, *p* = 0.1594; fecundity: one-way ANOVA: F_2,395_ = 7.196, *p* = 0.0009; Tukey's multiple comparison post hoc test: virgin ds*LacZ* versus mated ds*LacZ*, *p*<0.01; mated ds*LacZ* versus mated ds*MISO*, *p*<0.01; virgin ds*LacZ* versus mated ds*MISO*, *p*>0.05). One, two, and three asterisks indicate *p*<0.05, *p*<0.01, and *p*<0.001, respectively.
(DOCX)

Table S2 **Oocyte length in mated ds** ***MISO*** **females compared to virgin and mated ds** ***LacZ*** **controls after blood feeding.** Oocytes showing lipid accumulation (as estimated by Nile-Red) were measured in ovaries dissected from ds*MISO* or ds*LacZ* virgin or mated females at five points (12, 24, 36, 48, and 60 h) after blood feeding. Oocytes from ds*MISO* and virgin females are consistently smaller than oocytes from ds*LacZ* females throughout development, and the three groups reach the same size only at 60 hpm (one-way ANOVA: 12 h, F_2,303_ = 10.84, *p*<0.0001; 24 h, F_2,297_ = 132.0, *p*<0.0001; 36 h, F_2,223_ = 169.2, *p*<0.0001; 48 h, F_2,106_ = 82.29, *p*<0.0001; 60 h, F_2,105_ = 1.024, *p* = 0.03627). At each time point, means with different letters are significantly different (Tukey's multiple comparison post hoc test: *p*<0.001).
(DOCX)

Table S3 **List of primers and concentrations used for qRT-PCR.**
(DOCX)

## Abbreviations

20E: 20-hydroxy-ecdysone
EcR: Ecdysone receptor
HR3: Hormone receptor 3
JH: Juvenile Hormone
Lp: Lipophorin
MAGs: male accessory glands
USP: Ultraspiracle
Vg: Vitellogenin
YPP: yolk protein precursor
