# Supplementary figures and images for: The Interaction between a Sexually Transferred Steroid Hormone and a Female Protein Regulates Oogenesis in the Malaria Mosquito Anopheles gambiae
Source: PLoS Biol. 2013 Oct 29;11(10):e1001695. doi: 10.1371/journal.pbio.1001695 (PMC3812110; doi:10.1371/journal.pbio.1001695)

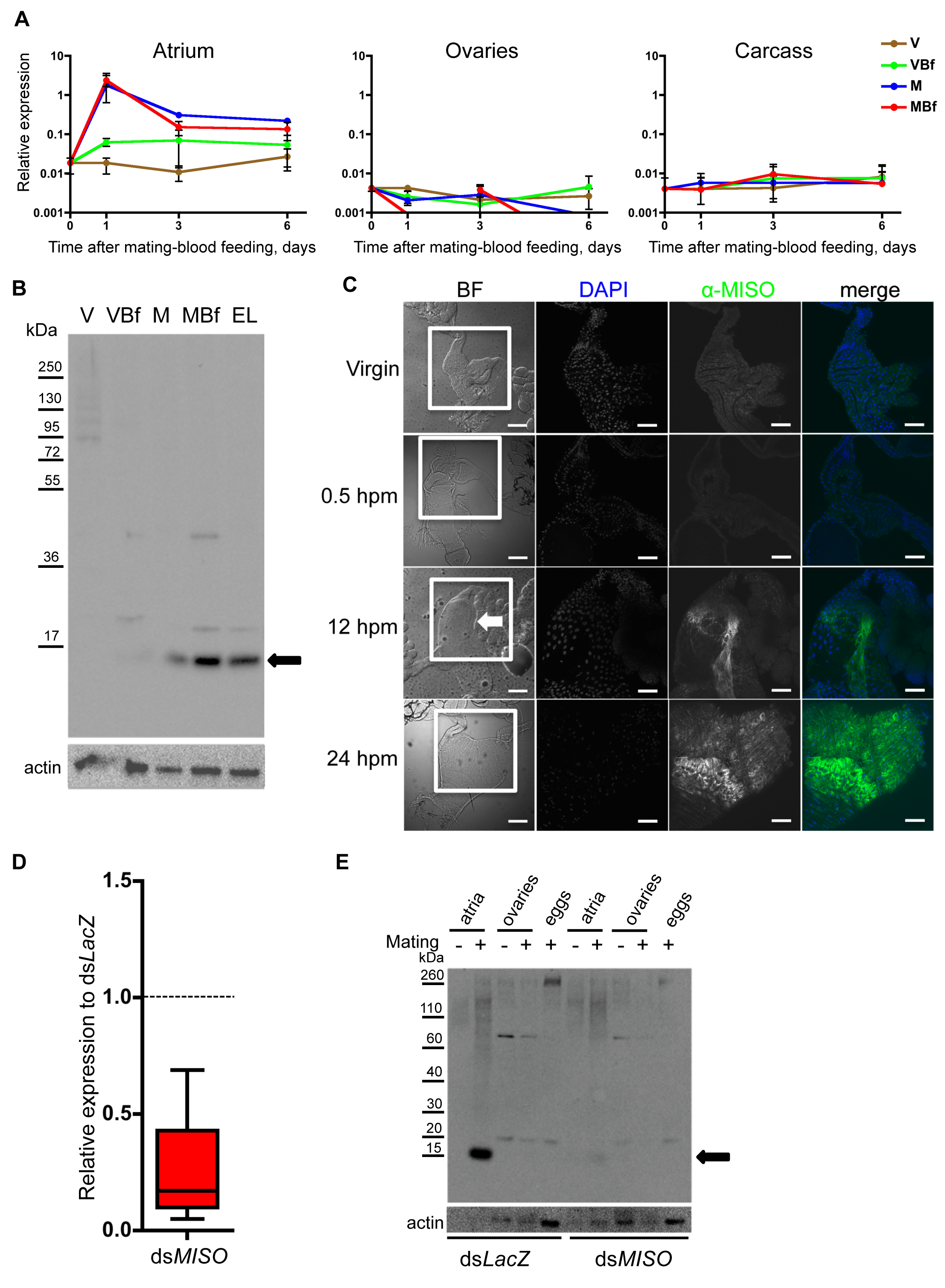

Supplement: Figure S1 — MISO is strongly induced in the atrium after mating and is secreted in the ampullae. (A) Quantitative RT-PCR (qRT-PCR) showing MISO expression in three conditions: virgin females after a blood meal (VBf), mated females (M), and mated females that have been blood fed immediately after mating (MBf). Atria, ovaries, and the rest of the body (carcass) were analyzed at different days (1, 3, and 6 d) postmating and/or blood feeding, and in age-matched virgin females. Expression levels (shown in logarithmic scale) were normalized to the housekeeping gene RpL19. The analysis was performed in three replicates on pools of 5–10 tissues, and data are represented as mean ± SEM. (B) Immunoblot analysis of MISO using a polyclonal antibody raised against a peptide fragment of the protein. Atria were dissected from different groups of females: virgins (V); mated (M) at 24 hpm; virgin blood fed (VBf) dissected at 24 h post-blood-feeding; mated blood fed (MBf), dissected at 24 h postmating and blood feeding; and MBf dissected after egg laying (EL). Immunoreactive bands (arrow) corresponding to the predicted 15 kDa size of MISO were detected in M, MBf, and EL atria. Actin was used as loading control. (C) Confocal analysis of MISO (green) in the atrium of virgin and mated females. The images next to the bright field (BF, scale bar: 100 µm) are magnifications (xy section, scale bar: 50 µm) of the regions indicated in the inset. At 12 hpm the mating plug is visible in the atrium (arrowhead). Cell nuclei (blue) are labeled with DAPI. (D) cDNAs from 15 independent replicates of dsMISO injections in virgin females analyzed by qRT-PCR at 24 hpm. RpL19 relative expression levels were compared between dsMISO- and dsLacZ-injected females (dotted line). Data are represented as a box-and-whisker diagram. (E) Immunoblot analysis of the efficacy of MISO silencing in protein extracts from atria, ovaries, and eggs. Atria and ovaries were dissected from virgin or mated females at 24 hpm that were injec [file pbio.1001695.s001.tif]

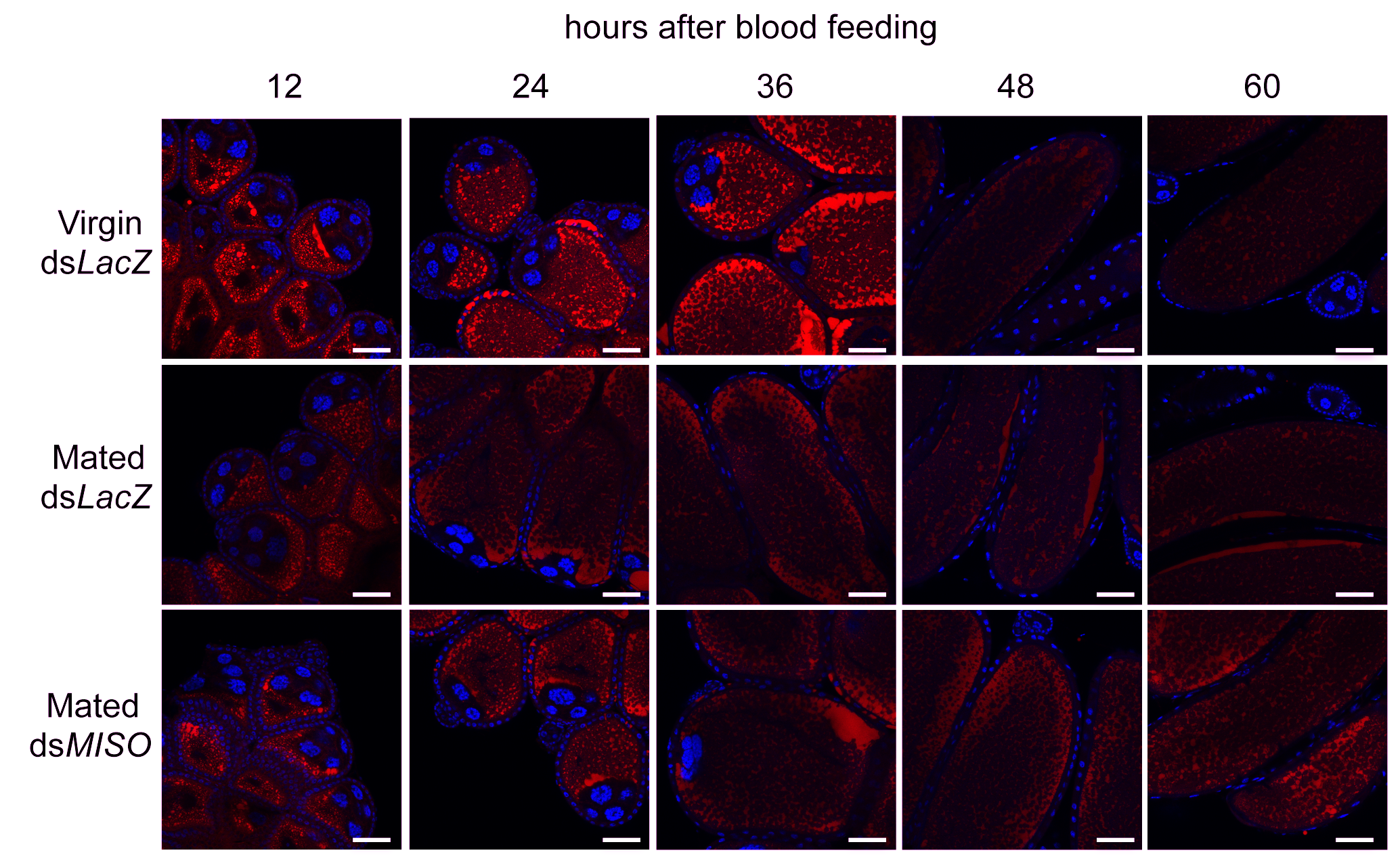

Supplement: Figure S2 — MISO silencing induces a delay in ovarian development. Immunofluorescence of oocyte development in ovaries dissected from dsMISO or dsLacZ-injected virgin or mated females at five points (12, 24, 36, 48, and 60 h) after blood feeding. Nile-Red (red) and DAPI (blue) were used to stain lipids and cell nuclei, respectively. Scale bar: 50 µm. (TIF) [file pbio.1001695.s002.tif]

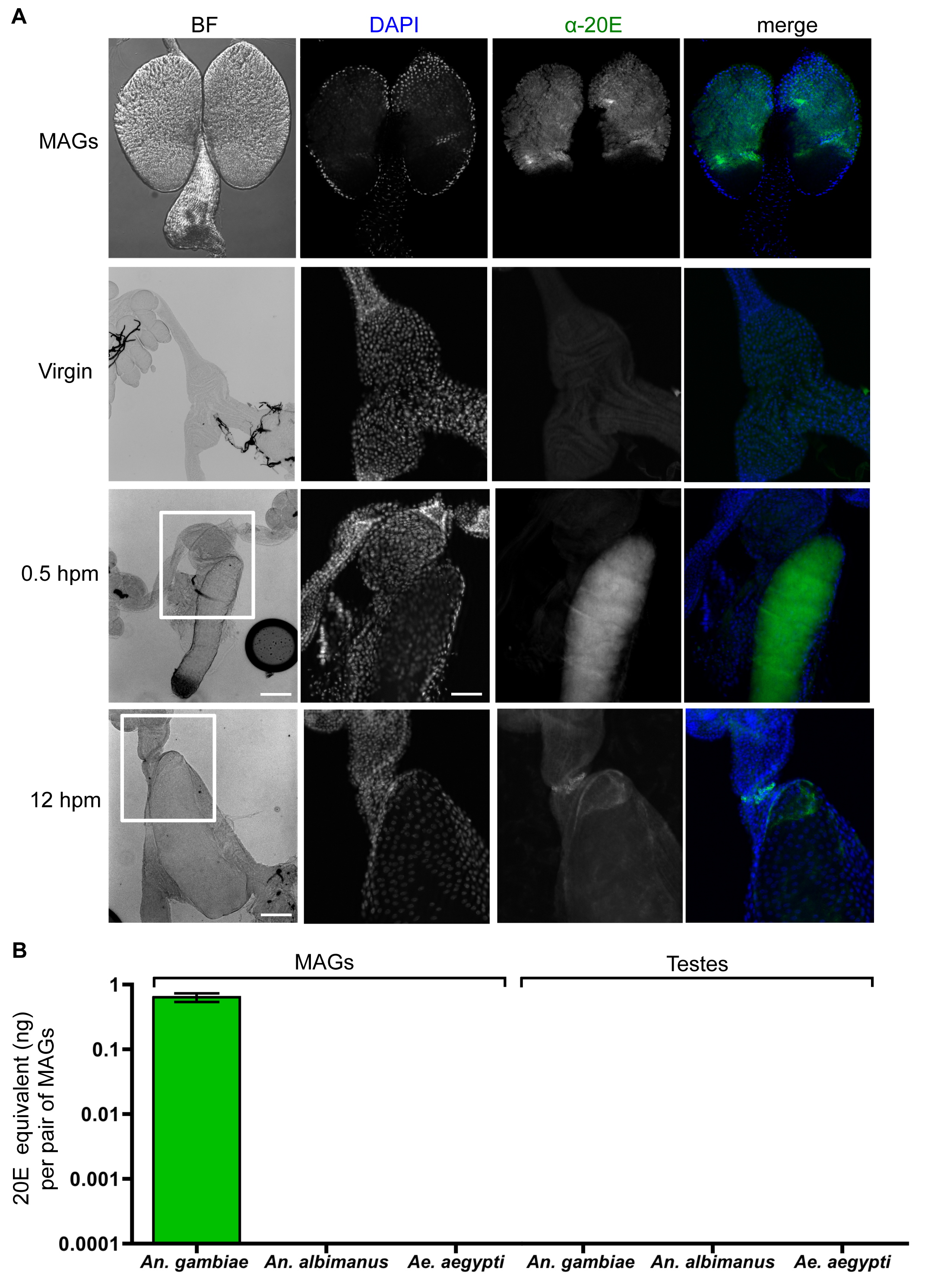

Supplement: Figure S3 — 20E localization in MAGs and atrium and quantification in male reproductive tracts from three mosquito species. (A) MAGs dissected from virgin males (MAGs) and atria dissected from virgin (V) and mated females at two time points after mating (0.5 hpm and 12 hpm) were dissected and incubated with anti-20E antibody (green). Cell nuclei (blue) are labeled with DAPI. Scale bar of the bright field (BF): 100 µm. The images next to the bright field (BF) are a magnification (xy section) of the region indicated by the inset (scale bar: 50 µm). (B) ELISA quantifications of 20E levels in MAGs and testes from either A. gambiae, A. albimanus, or A. aegypti males. A pool of 10 tissues was used for each of three replicates. Data are represented as mean ± SEM. (TIF) [file pbio.1001695.s003.tif]

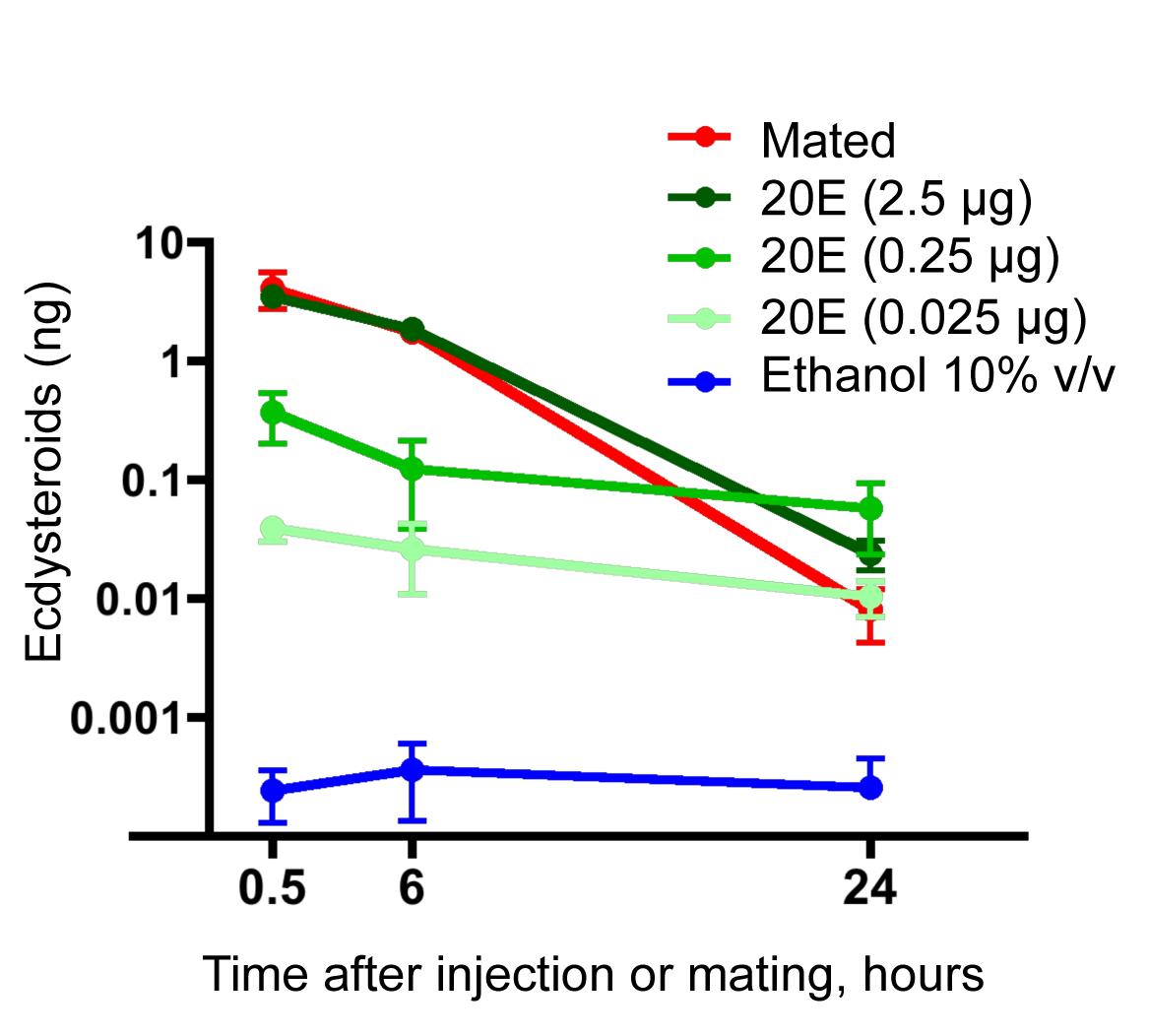

Supplement: Figure S4 — 20E quantification in the atrium after injection. ELISA quantification of 20E levels in female atria was performed prior or post injection (at 0.5 h, 6 h, and 24 h postinjection) of different 20E dilutions in the hemolymph of virgin females, or at the same time points after mating. Three 1∶10 dilutions starting from 2.5 µg per mosquito were injected. Ethanol injections were used as a control. A pool of 10 atria was used for each of three replicates. Data are represented as mean ± SEM. (TIF) [file pbio.1001695.s004.tif]
