# Supplementary material for: The Interaction between a Sexually Transferred Steroid Hormone and a Female Protein Regulates Oogenesis in the Malaria Mosquito Anopheles gambiae
Source: PLoS Biol. 2013 Oct 29;11(10):e1001695. doi: 10.1371/journal.pbio.1001695 (PMC3812110; doi:10.1371/journal.pbio.1001695)
Supplement: Table S1 — Summary of phenotypic analysis of ds MISO -injected females. MISO knockdown results in higher proportion of females that fail to develop eggs in both the oviposition and the egg development (oogenesis) assay (dsLacZ mated versus dsMISO mated: χ 2 = 6.864, p = 0.0088; dsLacZ mated versus dsLacZ virgin: χ 2 = 3.553, p = 0.0594). Among females that completed oogenesis, injections of dsMISO reduced the number of developed eggs (oviposition: t test: t219 = 0.9994, p = 0.1594; fecundity: one-way ANOVA: F2,395 = 7.196, p = 0.0009; Tukey's multiple comparison post hoc test: virgin dsLacZ versus mated dsLacZ, p<0.01; mated dsLacZ versus mated dsMISO, p<0.01; virgin dsLacZ versus mated dsMISO, p>0.05). One, two, and three asterisks indicate p<0.05, p<0.01, and p<0.001, respectively. (DOCX) [file pbio.1001695.s005.docx]

| **Experiment** | **dsRNA** | **mating status** | **n. repl.** | **n. examined** | **no egg laying (%)** | **no eggs (%)** | **n. eggs ( ± SD)** |
| --- | --- | --- | --- | --- | --- | --- | --- |
| oviposition | ds*LacZ* | mated | 3 | 138 | 13 (9.4) | 5 (3.6) | 91.1 (44.2) |
| oviposition | ds*MISO* | mated | 3 | 125 | 29 (23.3) ** | 20 (16) *** | 85.1 (43.5) |
| oogenesis | ds*LacZ* | mated | 6 | 147 | n.a. | 7 (4.8) | 81.7 (31.2) |
| oogenesis | ds*MISO* | mated | 6 | 164 | n.a. | 22 (13.4)** | 70.2 (29.8) ** |
| oogenesis | ds*LacZ* | virgin | 6 | 130 | n.a. | 14 (10.8) ≈* | 69.8 (26.1) ** |
