# Supplementary material for: The Interaction between a Sexually Transferred Steroid Hormone and a Female Protein Regulates Oogenesis in the Malaria Mosquito Anopheles gambiae
Source: PLoS Biol. 2013 Oct 29;11(10):e1001695. doi: 10.1371/journal.pbio.1001695 (PMC3812110; doi:10.1371/journal.pbio.1001695)
Supplement: Table S2 — Oocyte length in mated ds MISO females compared to virgin and mated ds LacZ controls after blood feeding. Oocytes showing lipid accumulation (as estimated by Nile-Red) were measured in ovaries dissected from dsMISO or dsLacZ virgin or mated females at five points (12, 24, 36, 48, and 60 h) after blood feeding. Oocytes from dsMISO and virgin females are consistently smaller than oocytes from dsLacZ females throughout development, and the three groups reach the same size only at 60 hpm (one-way ANOVA: 12 h, F2,303 = 10.84, p<0.0001; 24 h, F2,297 = 132.0, p<0.0001; 36 h, F2,223 = 169.2, p<0.0001; 48 h, F2,106 = 82.29, p<0.0001; 60 h, F2,105 = 1.024, p = 0.03627). At each time point, means with different letters are significantly different (Tukey's multiple comparison post hoc test: p<0.001). (DOCX) [file pbio.1001695.s006.docx]

| **Sample** | **Oocyte mean length μm (±SD)** | | | | |
| --- | --- | --- | --- | --- | --- |
|  | **12 h** | **24 h** | **36 h** | **48 h** | **60 h** |
| Mated ds*LacZ* | 121 (13) ^a^ | 174 (22) ^a^ | 331 (39) ^a^ | 453 (21) ^a^ | 498 (24) ^a^ |
| Virgin ds*LacZ* | 118 (10) ^a^ | 131 (13) ^b^ | 227 (41) ^b^ | 411 (26) ^b^ | 492 (21) ^a^ |
| Mated ds*MISO* | 112 (16) ^b^ | 142 (19) ^c^ | 256 (27) ^c^ | 376 (28) ^c^ | 491 (16) ^a^ |
