# Supplementary material for: The Interaction between a Sexually Transferred Steroid Hormone and a Female Protein Regulates Oogenesis in the Malaria Mosquito Anopheles gambiae
Source: PLoS Biol. 2013 Oct 29;11(10):e1001695. doi: 10.1371/journal.pbio.1001695 (PMC3812110; doi:10.1371/journal.pbio.1001695)
Supplement: Table S3 — List of primers and concentrations used for qRT-PCR. (DOCX) [file pbio.1001695.s007.docx]

| **Gene ID** | **FWD-REV sequence (5’3’)** | **nM** |
| --- | --- | --- |
| AGAP002620  (MISO) | AGACGATGGAGGGACTGATG  GGATTCGCTTTCGTGCTG | 300  300 |
| AGAP004203  (Vg) | CCGACTACGACCAGGACTTC  CTTCCGGCGTAGTAGACGAA | 300  300 |
| AGAP001826  (Lp) | CAGCCAGGATGGTGAGCTTAA  CACCAGCACCTTGGCGTT | 300  300 |
| AGAP009584 | AAGATCGTCGTGGTGAAGGT  CGCTTGATGAACAGGAAGGT | 300  300 |
| AGAP012211  (EcR) | CGTCGCTCACAGATGCATACAG  TCGACGGGGACAAATCTTCACG | 300  300 |
| AGAP009002  (HR3) | AATGGCGTACGAGGAAACAC  GAAAACGTACTGCGGGTGAT | 300  300 |
| AGAP002095  (USP) | AGAAGGAGAAACCGATGCTG  AAATGTCCGGCTTCAGGTC | 300  300 |
